# Bilateral Peritonsillar Abscesses Subsequent to Coronavirus Disease 2019 (COVID-19): A Case Report

**DOI:** 10.7759/cureus.78590

**Published:** 2025-02-05

**Authors:** Hisataka Ominato, Takumi Kumai, Ryo Ota, Miki Takahara

**Affiliations:** 1 Department of Otolaryngology, Japan Community Health Care Organization Hokkaido Hospital, Sapporo, JPN; 2 Department of Otolaryngology and Head and Neck Surgery, Asahikawa Medical University, Asahikawa, JPN

**Keywords:** bilateral peritonsillar abscesses, covid-19, fusobacterium species, head and neck infections, secondary bacterial infections

## Abstract

Superinfections with various bacteria exacerbate the symptoms of coronavirus disease 2019 (COVID-19). Bilateral peritonsillar abscesses are rare and could be fatal, and their occurrence after COVID-19 has not been reported.

A 20-year-old woman visited our hospital with a sore throat. The patient was diagnosed with COVID-19 infection, with no tonsillitis or peritonsillar abscess. Five days later, she returned to our department because of the deterioration of a sore throat. The patient was diagnosed with bilateral peritonsillar abscesses and admitted to the hospital for abscess drainage and antibiotics, which relieved her symptoms in seven days.

Although a sore throat is a common symptom of COVID-19, clinicians should be aware of the possibility of other upper airway diseases, including bilateral peritonsillar abscess, during COVID-19 treatment.

## Introduction

The recent global coronavirus disease 2019 (COVID-19) pandemic has significantly impacted our lives. Patients with COVID-19 have been reported to experience superinfection with various bacteria [1]. Among superinfections, upper airway infections such as epiglottitis could be fatal by inducing suffocation [2]. Tonsillitis is a common upper airway infection that could form a peritonsillar abscess. Peritonsillar abscess is the accumulation of pus in the fibrous capsule between the tonsil and superior pharyngeal constrictor muscle. Although the symptoms of peritonsillar abscesses are similar to those of viral pharyngitis and acute tonsillitis, abscess drainage and antibiotics are essential to treat this disease. Peritonsillar abscesses are usually unilateral; bilateral cases are rare [3]. Unlike unilateral peritonsillar abscesses, bilateral peritonsillar abscesses are relatively difficult to diagnose because of the bilateral symmetry of oral findings. Bilateral peritonsillar abscesses occurring as superinfection of COVID-19 have not been reported. Here, we report a case of bilateral peritonsillar abscess after COVID-19.

## Case presentation

**Figure 1:**
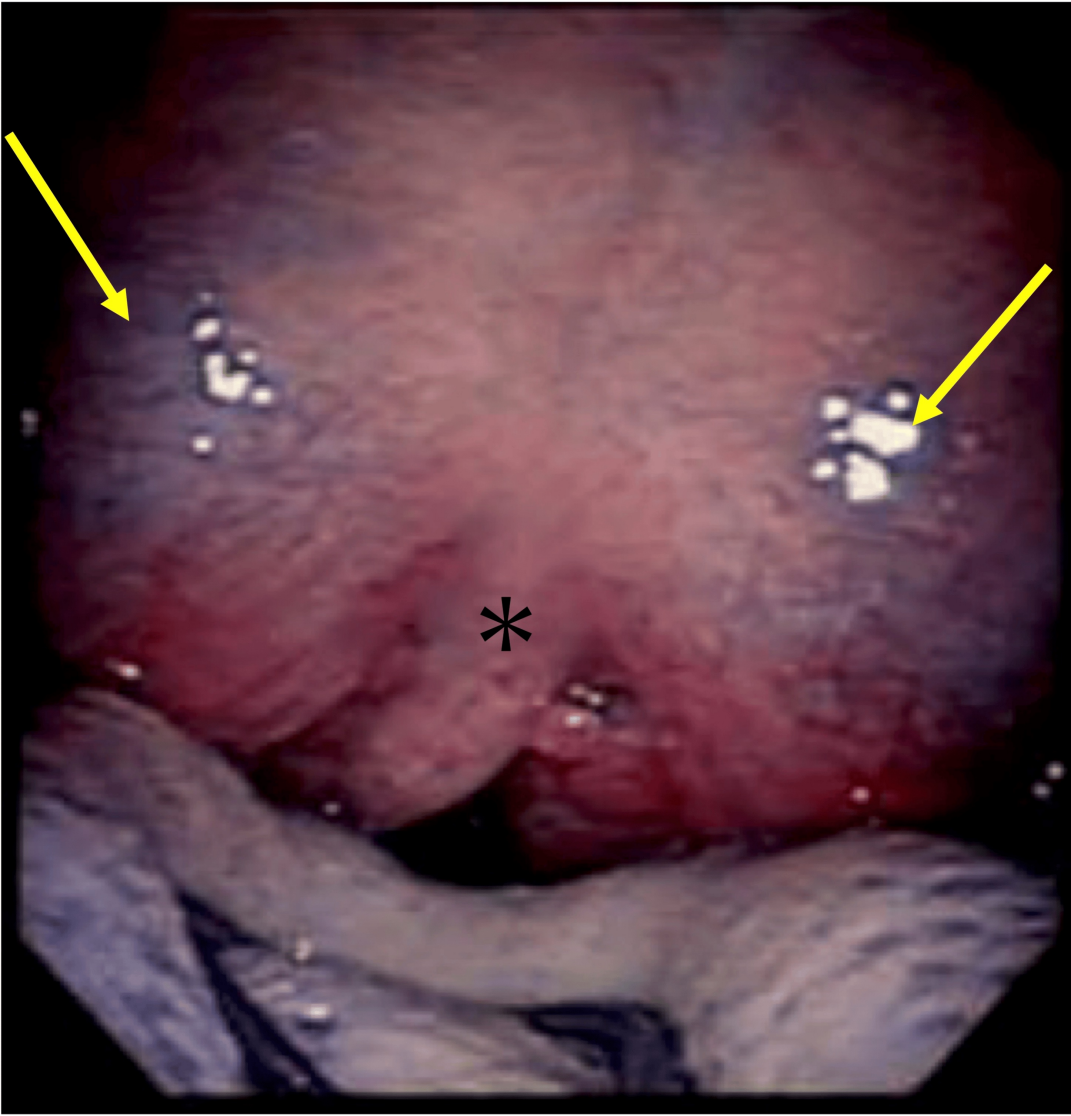
Bilateral peritonsillar swelling Photographic image of bilateral tonsils and bilateral peritonsillar swelling (arrow) *: uvula

A 20-year-old woman visited our hospital with a sore throat. The patient had no medical history including immunodeficiency. She had received four doses of mRNA vaccine against severe acute respiratory syndrome coronavirus 2 (SARS‑CoV‑2). Physical examination revealed redness of the bilateral posterior pharyngeal wall without tonsillitis. COVID-19 antigen test using nasal swabs revealed positive. The patient only received symptomatic therapy without antibiotics. Five days later, the patient returned to our department because of the exacerbation of sore throat, limited mouth opening (trismus), and fever (maximum 39℃). Physical examination revealed bilateral tonsillar and peritonsillar swelling (Figure 1).

Endoscopic examination revealed no laryngeal edema. Laboratory tests showed a peripheral white blood cell count of 11,310/μl and a C-reactive protein level of 4.31 mg/dl. The results of other laboratory tests are shown in Table 1.

**Table 1:** Laboratory data

	Hospitalization		Reference range
	Day 0	Day 4	
Complete blood cell count			
White blood cell count (/μl)	11310	5890	4000-10000
Neutrophils (%)	67.5	30.5	40.0-70.0
Lymphocytes (%)	25.5	62.0	20.0-55.0
Red blood cell count (×10^4^/μl)	475	386	380-480
Hemoglobin (g/dl)	13.6	10.9	11.0-16.0
Platelet count (×10^4^/μl)	34.6	24.4	15.0-40.0
Blood chemistry			
Total protein (g/dl)	8.8	5.8	6.0-8.0
Albumin (g/dl)	4.4	2.9	3.9-4.9
Aspartate aminotransferase (U/l)	18	13	6-40
Alanine aminotransferase (U/l)	12	9	6-37
Lactate dehydrogenase (U/l)	212	106	105-229
Blood urea nitrogen (mg/dl)	16.9	8.8	6.0-20.9
Creatinine (mg/dl)	0.58	0.47	0.4-0.8
Sodium (mEq/L)	140	141	133-145
Potassium (mEq/L)	4	3.4	3.4-4.7
Chloride (mEq/L)	100	102	98-108
Glucose (mg/dl)	104	-	70-100
Hemoglobin A1C (%)	5.6	-	4.6-6.2
C-reactive protein (mg/dl)	4.31	0.86	0-0.3

**Figure 2:**
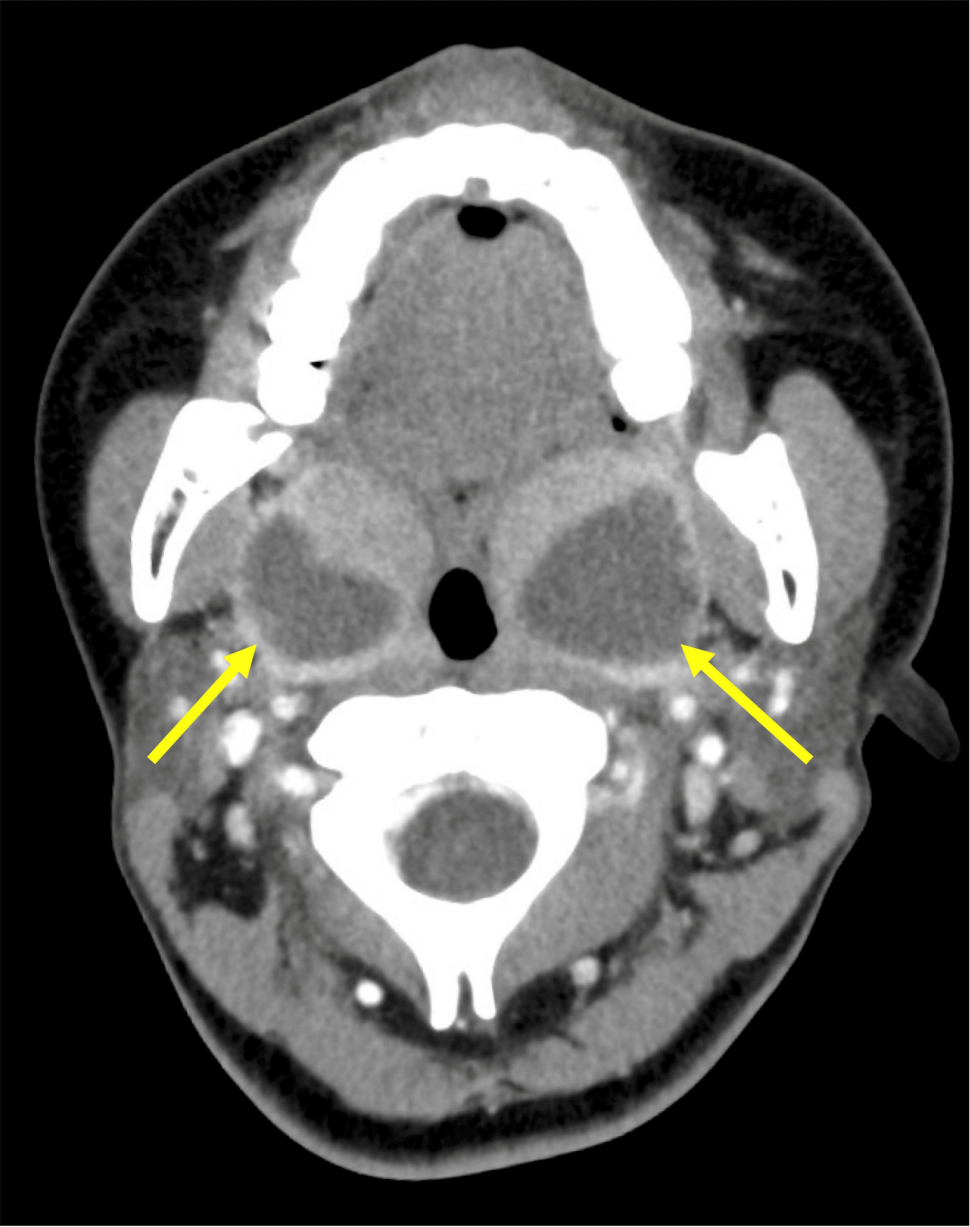
CT findings of bilateral peritonsillar abscess CT showing bilateral peritonsillar abscess (arrow) CT: computed tomography

Contrast-enhanced computed tomography (CT) scan revealed bilateral peritonsillar abscesses (Figure 2).

After hospitalization, pus was aspirated from the bilateral peritonsillar spaces, and incisions were made at the bilateral sites of aspiration for drainage. The patient was treated with ampicillin-sulbactam 9 g IV for seven days, and the symptoms immediately improved with near-normal levels of inflammatory responses (Table 1). Bacterial cultures from the pus showed the presence of *Fusobacterium *species and *Streptococcus mitis/oralis*, both of which were susceptible to ampicillin-sulbactam (Table 2).

**Table 2:** Antimicrobial susceptibility testing MIC: minimum inhibitory concentration; I: intermediate; R: resistant; S: susceptible

Antimicrobial agents	MIC (mg/L) and interpretation of susceptibility	Interpretation of susceptibility
	Streptococcus mitis/oralis	*Fusobacterium *species
Penicillin G	0.25 (S)	
Ampicillin	0.25 (S)	
Ampicillin-sulbactam	<0.25 (S)	S
Cefditoren pivoxil		S
Cefmetazole		S
Cefotaxime	0.5 (S)	
Ceftriaxone	0.25 (S)	I
Cefozopran	0.5 (S)	
Cefepime	<0.5 (S)	
Cefoperazone-sulbactam		S
Meropenem	0.25 (S)	
Rifampicin	>2 (R)	R
Clindamycin	>1 (R)	S
Minocycline	>4 (R)	
Chloramphenicol	<4 (S)	
Vancomycin	0.5 (S)	
Levofloxacin	>8 (R)	R

The patient was discharged with oral amoxicillin/clavulanate for five days without any signs of relapse.

## Discussion

The COVID-19 pandemic has changed the environment for diagnosing and treating upper airway diseases. During the pandemic, the diagnosis of many upper airway cases was delayed because of the restriction of oral procedures to prevent the risk of aerosol generation [4]. The mechanisms underlying simultaneous COVID-19 and bacterial infection remain unclear [5]. In the present case, bacterial infection after COVID-19 would induce the peritonsillar abscess. One potential explanation is that COVID-19-induced immune suppression rendered the patient susceptible to secondary bacterial infections. Previous studies have reported that COVID-19 can lead to lymphopenia, impaired T-cell function, and dysregulation of innate immunity [6,7]. Suppression of the immune response and disruption of tight junctions are reported to occur specifically in the early stages of COVID-19 [8], all of which contribute to an increased risk of bacterial infection. In addition, SARS-CoV-2 remains in the mucous membranes of the pharynx after acute infection followed by chronic inflammation [9], which might render the bacteria to invade through the damaged mucous membrane barrier.

The annual population incidence of peritonsillar abscess is considered one in 10,000/year [10]. Adequate treatments with antimicrobial therapy and surgical drainage of the abscess are necessary to prevent airway obstruction, abscess spreading including descending mediastinitis, and Lemierre's syndrome [11]. Surgical drainage should be considered in patients with advanced symptoms such as muffled voice, drooling, trismus, and dysphagia [12]. Peritonsillar abscesses are mostly unilateral and easy to diagnose by physical examination. Simultaneous infection with COVID-19 and unilateral peritonsillar abscess has been reported [5]. However, bilateral peritonsillar abscesses, which account for 3% of peritonsillar abscesses [13], could be overlooked by physical examination and have not been reported as superinfection with COVID-19. Because the symptoms of bilateral peritonsillar abscesses such as sore throat and fever are similar to those of COVID-19, the superinfection of these diseases may be difficult to diagnose in some cases. Since the tonsils were intact at the initial examination, the peritonsillar abscesses appeared after COVID-19 in our case. To promptly diagnose and treat superinfection with COVID-19, clinicians should recommend an early visit to the hospital if the symptoms have not been resolved with treatments against COVID-19. Procalcitonin would be a biomarker to detect bacterial superinfection with COVID-19 [14]. A contrast-enhanced CT scan may also help differentiate peritonsillar abscesses from other diseases, including infectious mononucleosis and malignant lymphoma [11]. Although the reported case of unilateral peritonsillar abscess with COVID-19 was treated with antimicrobial treatment alone [5], surgical drainage should be considered to secure the airway in severe cases including bilateral abscesses.

The relationship between peritonsillar abscesses and *Fusobacterium* species, particularly *F. nucleatum* and *F. necrophorum*, has been suggested as in our case. Klasinc et al. have reported that *F. necrophorum* elicits a deep neck infection including tonsillar and peritonsillar abscesses followed by Lemierre's syndrome and the head and neck region including the oral cavity and upper respiratory tract is the most common site for this infection [15]. It should be noted that the antibiotic susceptibility profile of *F. necrophorum* is 100% for amoxicillin and clavulanic acid [15] as in our case. Since the abundance of *Fusobacterium* species decreases in patients with COVID-19 [16], it is difficult to conclude whether COVID-19 can increase the incidence of *Fusobacterium* species infection from this single case report. Further accumulation of cases is required to confirm this issue and motivate clinicians to consider superinfection of the upper airway when the symptoms of COVID-19 have not improved.

## Conclusions

This article is the first report of bilateral peritonsillar abscesses as superinfection with COVID-19. CT scan is useful to detect bilateral peritonsillar abscesses. Since abscesses in the head and neck region associated with *Fusobacterium *species could occur in patients with COVID-19, clinicians should be aware of upper airway superinfection for timely management, including pus drainage and antibiotics.
